# Validation of maternal and terminal sheep breeding objectives using Irish field data

**DOI:** 10.1093/tas/txac099

**Published:** 2022-07-23

**Authors:** Noirin McHugh, Kevin McDermott, Alan Bohan, Lydia J Farrell, Jonathan Herron, Thierry Pabiou

**Affiliations:** Teagasc, Animal and Grassland Research and Innovation Centre, Moorepark, Fermoy, Co. Cork, Ireland; Sheep Ireland, Shinagh, Bandon, Co. Cork, Ireland; Sheep Ireland, Shinagh, Bandon, Co. Cork, Ireland; Teagasc, Animal and Grassland Research and Innovation Centre, Athenry, Co. Galway, Ireland; Teagasc, Animal and Grassland Research and Innovation Centre, Moorepark, Fermoy, Co. Cork, Ireland; Sheep Ireland, Shinagh, Bandon, Co. Cork, Ireland

**Keywords:** breeding objectives, genetic, maternal, sheep, validation

## Abstract

Genetic evaluations provide producers with a tool to aid in breeding decisions and highlight the increase in performance achievable at the farm level through genetic gain. Despite this, large-scale validation of sheep breeding objectives using field data is lacking in the scientific literature. The objective of the present study was to evaluate the phenotypic differences for a range of economically important traits for animals divergent in genetic merit for the Irish national maternal and terminal sheep breeding objectives. A dataset of 17,356 crossbred ewes and 54,322 progeny differing in their maternal and terminal breeding index recorded in 139 commercial flocks was available. The association of the maternal index of the ewe or terminal index of the ram and a range of phenotypic performance traits, including lambing, lamb performance, ewe performance, and health traits, were undertaken. Ewes excelling on the maternal index had higher litter sizes and produced progeny with greater perinatal lamb survival, heavier live weights from birth to postweaning and reduced days to slaughter (*P* < 0.05). Ewe maternal index had no quantifiable impact on lambing ease, carcass conformation, or fat, the health status of the ewe or lamb, ewe barren rate, or ewe live weight. Lambs born to rams of superior terminal index produced heavier lambs from preweaning onwards, with a reduced day to slaughter (*P* < 0.05). Lambing traits, lamb health, and carcass characteristics of the progeny did not differ between sires stratified as low or high on the terminal index (*P* > 0.05). Results from this study highlight that selecting either ewes or rams of superior maternal or terminal attributes will result in an improvement on pertinent performance traits of the national sheep flock, resulting in greater flock productivity and profitability.

## INTRODUCTION

Sheep systems are traditionally operated as low input with low-profit margins, therefore, strategies are required to improve farm sustainability and profitability (Bohan et al., 2016); the use of national genetic indexes is one such strategy. Breeding objectives provide producers with a tool to aid in breeding decisions and highlight the increase in performance achievable at farm level through genetic selection. Breeding objectives in sheep and beef cattle are generally represented as maternal (reproduction or ewe trait-based) or terminal (growth and meat trait-based) or combinations of both (Swan et al., 2007; Byrne et al., 2012); dual sheep breeding objectives have been defined previously for the Irish sheep industry (Santos et al., 2015; Bohan et al., 2019). Genetic gain, however, remains a function of how accurately the breeding objectives reflect differences in the phenotypic performance of animals. Although validation of sheep breeding objectives has been reported previously, these tended to be performed under controlled experiments both in Ireland (Fetherstone et al., 2021a, 2021b) and elsewhere (Conington et al., 1998; Márquez et al., 2012, 2013). Large-scale validation of breeding objectives using field data in sheep is not often described in the scientific literature. Quantifying the mean performance difference for parents and their progeny divergent for a breeding objective is critical to ensure producer confidence in the national genetic evaluations and thus has the potential to increase the rates of genetic gain achievable. The objective of the present study, therefore, was to evaluate the phenotypic differences for a range of economically important traits for sheep divergent in genetic merit for the Irish national maternal and terminal indexes. Results from this study form an integral part in the validation of the maternal and terminal sheep breeding objectives in Ireland and highlight the expected benefits of adopting a genetic index approach to improve the performance of the national sheep flock across a plethora of traits.

## MATERIALS AND METHODS

### Phenotypic Data

Performance data from 139 commercial flocks across a range of animal-specific events including lambing (i.e., lambing difficulty, lamb survival, and birth weight); lamb performance (i.e., live weight and carcass data); ewe performance records (i.e., number of lambs born and ewe live weight); and animal health (i.e., dag score and lameness scores) recorded between October 2018 and June 2021 were extracted from the Sheep Ireland database (https://www.sheep.ie/). In total, performance data were available on 17,356 crossbred ewes and 54,322 progeny.

### Lambing Performance

Data were available on three producer-recorded lambing traits: birth weight, lambing difficulty score, and lamb survival as described by McHugh et al. (2016). Lamb birth weight was measured within 24 h of birth using weighing scales; only lambs with a birth weight between 2 and 9 kg were considered. Lambing difficulty score was subjectively scored by producers on a scale of 1 to 4 as: 1 = no lambing assistance; 2 = voluntary assistance; 3 = slight assistance; 4 = significant assistance (including caesarean). Within the Irish national genetic evaluation, the lambing difficulty scores are treated separately for single and multiple litters (McHugh et al., 2020) and further dichotomized into a lambing ease trait whereby ewes requiring no assistance/unobserved or voluntary assistance were coded as 1 and ewes requiring slight or severe assistance were coded as 0. Lamb survival was scored as whether the lamb was alive (survival = 1) or dead (survival = 0) within 24 h of birth. For all lambing traits, contemporary groups were defined as week of birth-flock of birth (McHugh et al., 2017); only contemporary groups with at least five lambs were retained.

### Lamb Performance

Lamb live weight was recorded at three time points: preweaning, weaning, and postweaning (McGovern et al., 2020) by producers using weigh scales. Preweaning weight was defined as the live weight taken between 20 and 65 days of age; only records of lambs weighing between 12 and 32 kg were retained. Weaning weight was defined as the live weight taken between 66 and 120 days of age; only records of lambs weighing between 20 and 50 kg were retained. Postweaning weight was defined as a lamb between 121 and 180 days of age and weighing between 25 and 65 kg. Lamb carcass data including date of slaughter, carcass weight, carcass conformation, and carcass fat were also available on a subset of lambs (*n* = 12,637) slaughtered between 60 and 365 days of age. For each weighing event, each lamb was allocated to a contemporary group of flock-date of weighing. For all carcass traits, lambs were allocated to a contemporary group of abattoir-date of slaughter. Across all lamb performance traits, only contemporary groups with at least five records were retained.

### Ewe Performance Traits

Ewe live weight was defined as the weight of a female who had at least one recorded lambing event; only recorded ewe live weights between 30 and 130 kg were retained (McHugh et al., 2019). Ewe litter size was defined as the number of lambs born per litter for each lambing event; only ewe litter sizes between one (singles) and four (quadruplets) were considered. Ewe barren rates were recorded by individual producers and was defined as the ewe was recorded as having lambed (barren = 0) or not (barren = 1) within a given production year. For ewe live weight, the contemporary group was defined as flock-by-date of weighing. For ewe litter size, the contemporary group was defined as flock-by-week of lambing. For ewe barren rate, the contemporary group was defined as flock-by-mating season. For all ewe performance traits, only contemporary groups with at least five records were retained for analysis.

### Health Traits

Dag score was measured by trained technicians on a five-point scale as 1 = fecal soiling and dags covering the breech area and extending down the hind legs towards the pasterns to 5 = no fecal soiling (O’Brien et al., 2017). Dag score was measured on animals between 40 and 180 days of age and generally coincidence with a weighing event. Lameness was measured on an incidence basis by trained technicians or producers by assessing each animal individually as it walked and each animal was recorded as being lame (lame = 1) or not (lame = 0) and was treated as a separate trait for ewes and lambs (O’Brien et al., 2017). For all health traits (i.e., dag score, ewe lameness, and lamb lameness), the contemporary group was defined as flock-by-date of health recording; only contemporary groups with at least five records were retained for analysis.

Across all traits, data were also available on dam age number (or ewe age for traits of the ewe herself). Ewe or dam age was classified into five categories as 1, 2, 3 (3 to 5), 4 (6 to 8), and 5 (≥9). Age of the ewe or dam at first lambing was categorized as lambing either: 1) between 8 and 18 months of age, or 2) between ≥ 18 and 28 months of age as per McHugh et al. (2016). The numbers of records available for each trait are shown in Table 1.

**Table 1.** Number of records (*n*), number of ewes, mean (µ; SD in parenthesis), and number of contemporary groups (CG) for each trait

Trait	*n*	Ewes	µ, SD	CG
Lambing				
Lambing difficulty score single, 1–4^1^	9,197	7,129	1.45 (0.79)	541
Lambing difficulty score multiple, 1–4^1^	37,972	11,257	1.52 (0.82)	589
Perinatal survival, %^4^	47,169	15,453	93.48 (24.69)	669
Birth Weight, kg	44,041	14,958	4.77 (1.12)	628
Lamb performance				
Preweaning weight, kg	27,909	10,619	18.95 (4.46)	236
Weaning weight, kg	30,296	10,945	32.08 (6.29)	289
Post weaning weight, kg	14,933	6,662	39.32 (6.59)	190
Carcass conformation, 1–5^2^	12,637	6,556	3.33 (0.50)	194
Carcass fat, 1–5^2^	12,637	6,556	2.90 (0.39)	194
Age at slaughter, d	12,637	6,556	195.04 (64.62)	194
Ewe performance				
Litter size, 1–4	17,627	6,090	1.83 (0.71)	823
Barren rate, %^4^	13,745	5,082	6.17 (24.07)	152
Mature weight, kg	12,022	4,379	71.30 (11.35)	76
Health				
Dag score, 1–5^3^	28,925	8,248	3.57 (0.82)	291
Lameness lamb, %^4^	35,654	7,334	5.23 (22.27)	357
Lameness ewe, %^4^	30,179	3,444	7.29 (26.00)	281

Score 1 (no assistance) to 4 (significant assistance).

Score 1 (poor conformation/low fat cover) to 5 (excellent conformation/high fat cover).

Score 1 (fecal soiling and dags covering the breech area) to 5 (no fecal soiling).

Traits are recorded on a scale of 1 (i.e., survived, barren or lame) or not (trait = 0). Traits were multiple by 100 to give percentage incidence for each trait.

### Validation Population

The maternal and terminal breeding indexes (expressed in monetary €uro’s; Bohan et al., 2019) for all central progeny test (CPT) animals from the October 2018 Irish genetic evaluations were available and used in the validation process. Therefore, the phenotypic data (described above) used to validate the genetic information did not contribute to the genetic evaluations. Sheep Ireland, the national body responsible for sheep genetic evaluations in Ireland, undertakes routine across-breed one-step genomic evaluations using MIX99 software (MiX99 Development Team, 2017) for four suites of traits, namely: lambing, lamb performance, ewe performance, and health (Pabiou et al., 2019). Approximately 50% of data used in the genetic evaluations originate from crossbred animals, therefore genetic evaluations were adjusted for the heterosis and recombination loss coefficient of the animal. Breed differences are accounted for by using fixed regressions in the model for the following breeds: Belclare, Beltex, Bluefaced Leicester, Charollais, Llyen, Suffolk, Rouge de l’Ouest, Texel, Vendeen; the remaining breeds were grouped as “other maternal” and “other terminal” depending on their main breeding orientation. The maternal and terminal index for each animal was computed for all animals using index weightings for the Irish national maternal and terminal index as described by Bohan et al. (2019) and shown in the Supplementary Appendix. The maternal and terminal index value for each animal was categorized separately into one of five stratus of equal size: High (top 20% of animals), Above average (top 60 to 80% of animals), Average (40 to 60% of animals), Below average (20 to 40%), and Low (0 to 20%).

### Data Analyses

The association of the maternal or terminal index and the phenotypic performance traits was quantified separately using a fixed effects model in SAS (SAS Institute, Cary, NC). For all models the index, either maternal or terminal, was considered on a categorical scale (i.e., High, Above average, Average, Below average, or Low).

For the lambing traits, the model employed was:


$$\begin{array}{ll} Y_{ijklmno}= & \;\mu +\;CG_{i}+\;EweAge_{j}+\;BirthType_{k}+\;Sex_{l}\\ & +\underset{m=1}{\overset{5}{\sum }}\;\beta_{m}Breed_{m}+\;EBV_{n}+\;Index_{o}+\;e_{ijklmno} \end{array}$$

where Y_*ijklmmo*_ is the dependent variable of lamb birth weight, lamb survival, or lambing ease; µ is the population mean; CG_*i*_ is the effect of the contemporary group (*i* = 1,…., 669); EweAge_*j*_ is the age category of the ewe (*j* = 1, 2, 3, 4, or 5); BirthType_*k*_ is the birth type of the litter (*k* = 1, 2, 3 or 4); Sex_*l*_ is the sex of the lamb (*l* = Male or Female); $\underset{l=m}{\overset{10}{\sum }}\;\beta_{m}Breed_{m}$ is the separate regression coefficients on the breed proportion of each of the 10 most recorded breeds (Belclare, Beltex, Bluefaced Leicester, Charollais, Llyen, Suffolk, Rouge de l’Ouest, Texel, Vendeen, and other); EBV was the covariate of the estimated breeding value (EBV) for the dependent variable of the litter’s sire (when the maternal index is under investigation) or dam (when the terminal index is under investigation); Index_*o*_ is the maternal index of the dam or the terminal index of the sire (*o* = High, Above average, Average, Below average, or Low); and e_*ijklmmo*_ is the residual term.

For lamb performance and health traits, the model employed was:


$$\begin{array}{l} \begin{array}{l} Y_{ijklmnopqrs}=\mu +\;CG_{i}+\;EweAge_{j}+\;BirthType_{k}\\ +\;RearType_{l}+\;Sex_{m}+\;Age_{n}+\underset{o=1}{\overset{5}{\sum }}\;\beta_{o}Breed_{o}+\\ \left(Month_{p}\right)+\left(CarcWt_{q}\right)\;+\;EBV_{r}+\;Index_{s}+\;e_{ijklmnopqrs} \end{array} \end{array}$$

where Y_*ijklmmopqrs*_ is the dependent variable of lamb weight (preweaning, weaning, or postweaning); carcass (conformation, fat, or age at slaughter) or health (lamb lameness and dag score) trait; µ is the population mean; CG_*i*_ is the effect of contemporary group (*i* = 1,..., 289); EweAge_*j*_ is the age category of the ewe (*j* = 1, 2, 3, 4, or 5); BirthType_*k*_ is the birth type of the litter (*k* = 1, 2, 3 or 4); RearType_*l*_ is the rearing type of the litter (*l* = 1, 2, or 3); Sex_*m*_ is the sex of the lamb (*m* = Male or Female); Age_*n*_ is the age of the lamb at recording (*n* = 20,…,365); $\underset{l=o}{\overset{10}{\sum }}\;\beta_{o}Breed_{o}$ is the separate regression coefficients on the breed proportion of each of the 10 breeds (Belclare, Beltex, Bluefaced Leicester, Charollais, Llyen, Suffolk, Rouge de l’Ouest, Texel, Vendeen, and other); EBV_*r*_ is the covariate of the EBV for the dependent variable of the lamb’s sire (when the maternal index is under investigation) or dam (when the terminal index is under investigation); and Index_*s*_ is the maternal index of the dam or the terminal index of the sire (*s* = High, Above average, Average, Below average, or Low). When the dependent variable under investigation was carcass conformation, fat, or age at slaughter the model also included: Month_*p*_ is the month of birth (*p* = January to May) and CarcWt_*q*_ is the covariate of actual carcass weight of the lamb. Across all models, e_*ijklmmopqrs*_ is the residual term.

For ewe traits, the model employed was:


$$\begin{array}{ll} Y_{ijklm}= & \;\mu +\;CG_{i}+\;EweAge_{j}+\;AFL_{k}\\ & +\underset{l=1}{\overset{5}{\sum }}\;\beta_{l}Breed_{l}+\;Index_{m}+\;e_{ijklm} \end{array}$$

where Y_*ijklm*_ is the dependent variable of ewe litter size, barren rate, ewe live weight, or ewe lameness score; µ is the population mean; CG_*i*_ is the effect of the contemporary group (*i* = 1,…, 291); EweAge_*j*_ is the age category of the ewe (*j* = 1, 2, 3, 4, or 5); AFL_*k*_ = age at first lambing (*k* = 8 and 18 months of age or ≥ 18 and 28 months of age); $\underset{l=1}{\overset{10}{\sum }}\;\beta_{l}Breed_{l}$ is the separate regression coefficients on the breed proportion of each of the 10 breeds (Belclare, Beltex, Bluefaced Leicester, Charollais, Llyen, Suffolk, Rouge de l’Ouest, Texel, Vendeen, and other); Index_*m*_ is the maternal index of the ewe (*m* = High, Above average, Average, Below average, or Low); and e_*ijklm*_ is the residual term.

## RESULTS

All 139 commercial flocks operated a spring lambing production system, with a median lambing date of 2nd April. The average lambing difficulty score was 1.45 and 1.52 in singleton and multiple litters, respectively (Table 1). The prevalence of perinatal lamb mortality was 6.52%. Lambs were weaned at an average of 101 days and weighed 32.08 (SD = 6.29) kg. The average age at slaughter was 195.04 (SD = 64.62) days. Across all flocks the average litter size was 1.83 (SD = 0.71; Table 1). The average weight of the ewe was 71.30 (SD = 11.35) kg. A greater incidence of lameness was recorded in ewes (7.29%) in comparison to lambs (5.23%; Table 1).

### Lambing *Traits*

Although lambing ease recorded in either singleton or multiple litters differed by the maternal index of the ewe (*P* < 0.05) no clear trend was observed across the five maternal index strata. The maximum difference in lambing ease observed between the five strata was 2.79% and 1.76% for single and multiple litters, respectively (Table 2). Greater perinatal lamb survival was associated with ewes with higher maternal indexes (*P* < 0.05), with a difference of 0.98% observed between ewes of the low and high maternal index strata. Greater lamb birth weights were associated with ewes of higher maternal indexes (Table 2).

**Table 2.** Mean maternal index value (€) for the five strata of maternal index of the ewe and the associated least square means (SE in parenthesis) for each lambing trait

Group	Maternal index, €	Lambing ease single, %	Lambing ease multiple, %	Perinatal survival, %	Birth weight, kg
Low	−€1.26	84.32 (3.66)^ab^	80.53 (1.12)^ab^	92.57 (0.66)^a^	4.24 (0.03)^a^
Below average	−€0.39	86.30 (3.66)^a^	80.31 (1.08)^ab^	93.14 (0.65)^ab^	4.29 (0.03)^b^
Average	€0.04	86.21 (3.60)^a^	81.41 (1.02)^a^	93.59 (0.62)^b^	4.31 (0.02)^b^
Above average	€0.69	83.51 (3.68)^b^	79.65 (1.06)^b^	93.38 (0.64)^b^	4.38 (0.03)^c^
High	€2.02	86.29 (3.66)^a^	80.97 (1.04)^a^	93.55 (0.63)^b^	4.37 (0.03)^c^

Values within columns with different superscripts differ (*P* < 0.05) from each other.

Lambing ease, perinatal lamb survival, and lamb birth weight all differed by the terminal index stratification of the ram (*P* < 0.05). However, across all the lambing traits no obvious trend was noted across sires stratified on terminal index, with no difference observed between sires stratified as low and high on terminal index for any of the lambing traits (Table 3).

**Table 3.** Mean terminal index value (€) for the five strata of terminal index of the sire and the associated least square means (SE in parenthesis) for each lambing trait

Group	Terminal index, €	Lambing ease single, %	Lambing ease multiple, %	Perinatal survival, %	Birth weight, kg
Low	−€0.70	85.94 (3.76)^ab^	79.48 (1.21) ^b^	94.07 (0.72)^a^	4.33 (0.03)^a^
Below Average	−€0.05	87.51 (3.74)^a^	85.71 (1.20) ^a^	92.98 (0.71)^b^	4.25 (0.03)^b^
Average	€0.26	85.70 (3.82)^ab^	79.54 (1.20) ^b^	93.38 (0.72)^ab^	4.38 (0.03)^a^
Above Average	€0.55	83.59 (3.75)^b^	81.23 (1.14) ^b^	93.51 (0.68)^ab^	4.33 (0.03)^a^
High	€1.27	85.59 (3.60)^ab^	80.37 (1.01)^b^	93.27 (0.62)^ab^	4.33 (0.02)^a^

Values within columns with different superscripts differ (*P* < 0.05) from each other.

### Lamb Performance *Traits*

Relative to ewes in the low maternal stratum, lambs from ewes in the high maternal stratum were heavier at preweaning (+0.39 kg), weaning (+0.80 kg), and postweaning (+0.99 kg) and had a shorter number of days to reach slaughter (−4.85 d; *P* < 0.05). Carcass fat did not differ by ewe maternal index (*P* > 0.05; Table 4). For carcass conformation small biological differences (+0.03 of a grade; *P* < 0.05) were observed between the ewe maternal index strata, but no clear trend was observed across the five strata.

**Table 4.** Mean maternal index value (€) for the five strata of matrnal index of the ewe and the associated least square means (SE in parenthesis) for each lamb performance trait.

Group	Maternal index, €	Preweaning weight, kg	Weaning weight, kg	Postweaning weight, kg	Carcass conformation, 1–5	Carcass fat, 1–5	Age at slaughter, d
Low	−€1.26	17.88 (0.17)^b^	31.07 (0.22)^d^	38.57 (0.35)^c^	3.45 (0.09)^a^	2.97 (0.07)	211.61 (9.82)^b^
Below average	−€0.39	17.96 (0.16)^b^	31.32 (0.22)^c^	39.05 (0.35)^b^	3.42 (0.09)^b^	2.97 (0.07)	210.51 (9.82)^b^
Average	€0.04	17.95 (0.16)^b^	31.36 (0.21)^c^	38.85 (0.33)^bc^	3.43 (0.09)^ab^	2.95 (0.07)	209.48 (9.76)^b^
Above average	€0.69	18.19 (0.16)^a^	31.55 (0.22)^b^	39.19 (0.34)^b^	3.44 (0.09)^ab^	2.96 (0.07)	208.18 (9.78)^ab^
High	€2.02	18.27 (0.16)^a^	31.87 (0.21)^a^	39.56 (0.34)^a^	3.45 (0.09)^a^	2.96 (0.07)	206.76 (9.79)^a^

Values within columns with different superscripts differ (*P* < 0.05) from each other.

Greater preweaning, weaning, and postweaning weights were associated with lamb born to rams of a higher terminal index (*P* < 0.05), and the weight difference between lambs born to rams in the low and high terminal index strata increased with lamb age (Table 5). Lambs born to rams in the high terminal stratum were slaughtered, on average, 8.34 d earlier than lambs born to rams in the low terminal stratum (*P* < 0.05). No difference was observed in the carcass fat score of lambs based on the terminal index of the sire (*P* > 0.05). Carcass conformation score tended to reduce based on a terminal index of the sire, with a 0.05 lower carcass conformation score associated with lambs born to sires in the high terminal index stratum relative to lambs of sires in the low terminal index stratum (Table 5; *P* < 0.05).

**Table 5.** Mean terminal index value (€) for the five strata of terminal index of the sire and the associated least square means (SE in parenthesis) for each lamb performance trait

Group	Terminal Index, €	Preweaning weight, kg	Weaning weight, kg	Postweaning weight, kg	Carcass conformation, 1–5	Carcass fat, 1–5	Age at slaughter, d
Low	−€0.70	17.85 (0.18)^b^	31.20 (0.23)^b^	38.65 (0.37)^b^	3.48 (0.09)^a^	2.98 (0.07)	215.09 (9.89)^c^
Below average	−€0.05	17.56 (0.17)^c^	30.49 (0.22)^c^	38.22 (0.36)^b^	3.46 (0.09)^ab^	2.97 (0.07)	217.89 (9.88)^c^
Average	€0.26	18.02 (0.17)^ab^	31.41 (0.22)^b^	39.10 (0.37)^ab^	3.47 (0.09)^ab^	2.97 (0.07)	209.41 (9.88)^ab^
Above average	€0.55	18.07 (0.17)^ab^	31.45 (0.21)^b^	39.18 (0.35)^a^	3.44 (0.09)^b^	2.95 (0.07)	210.06 (9.81)^b^
High	€1.27	18.15 (0.16)^a^	31.71 (0.19)^a^	39.35 (0.33)^a^	3.43 (0.09)^b^	2.96 (0.07)	206.75 (9.75)^a^

Values within columns with different superscripts differ (*P* < 0.05) from each other.

### Ewe Performance *Traits*

Litter size differed by ewe maternal index (*P* < 0.05), with a +0.17 greater litter size associated with ewes of high maternal index, relative to ewes of low maternal index (Table 6). Ewe barren rate did not differ by ewe maternal index (*P* > 0.05). Although differences were observed in ewe live weight across the five stratifications of ewe maternal index (*P* < 0.05), the differences were biologically small (ranging from 0.07 to 0.71 kg), and no clear trend was observed across the five strata for maternal index.

**Table 6.** Mean maternal index value (€) for the five strata of maternal index of the ewe and the associated least square means (SE in parenthesis) for each ewe performance trait

Group	Maternal index, €	Litter size, 1−4	Barren rate, %	Ewe weight, kg
Low	−€1.26	1.74 (0.03)^c^	-1.87 (0.46)	72.76 (0.51)^a^
Below average	−€0.39	1.74 (0.03)^c^	-2.07 (0.45)	72.69 (0.50)^a^
Average	€0.04	1.81 (0.03)^b^	-1.98 (0.45)	73.40 (0.49)^b^
Above average	€0.69	1.84 (0.03)^b^	-2.14 (0.44)	73.29 (0.48)^ab^
High	€2.02	1.91 (0.03)^a^	-1.99 (0.44)	73.29 (0.47)^ab^

Values within columns with different superscripts differ (*P* < 0.05) from each other.

### Health *Traits*

Dag score and ewe lameness differed by ewe maternal index (*P* < 0.05; Table 7), however, a clear trend was not detected between the five strata for either trait. The differences detected between the most divergent strata were +0.04 of a dag score and 1.25% for ewe lameness. Lameness recorded in lambs did not differ by ewe maternal index (*P* > 0.05).

**Table 7.** Mean maternal index value (€) for the five strata of maternal index of the ewe and the associated least square means (SE in parenthesis) for each health trait

Group	Maternal index, €	Dag score, 1–5	Lameness lamb, %	Lameness ewe, %
Low	−€1.26	3.80 (0.02)^a^	8.35 (0.58)	12.13 (2.15)^a^
Below average	−€0.39	3.84 (0.02)^b^	7.74 (0.56)	13.38 (2.15)^b^
Average	€0.04	3.80 (0.02)^a^	7.92 (0.54)	13.13 (2.14)^b^
Above average	€0.69	3.84 (0.02)^b^	8.11 (0.55)	12.61 (2.13)^a^
High	€2.02	3.82 (0.02)^a^	8.01 (0.54)	12.25 (2.13)^b^

Values within columns with different superscripts differ (*P* < 0.05) from each other.

Dag score differed by sire terminal index (*P* < 0.05; Table 8), however, similar to the ewe maternal index no obvious trend was noted between the strata, with small biological differences detected. Lameness recorded in lambs did not differ by ram terminal index (*P* > 0.05).

**Table 8.** Mean terminal index value (€) for the five strata of terminal index of the sire and the associated least square means (SE in parenthesis) for each health performance trait

Group	Terminal index, €	Dag score, 1–5	Lameness lamb, %
Low	−€0.70	3.82 (0.03)^b^	8.26 (0.69)
Below average	−€0.05	3.80 (0.03)^b^	7.39 (0.68)
Average	€0.26	3.77 (0.03)^a^	8.31 (0.67)
Above average	€0.55	3.84 (0.03)^c^	7.45 (0.63)
High	€1.27	3.82 (0.02)^b^	8.07 (0.51)

Values within columns with different superscripts differ (*P* < 0.05) from each other.

## DISCUSSION

Similar to other ruminant production systems, the profitability of sheep farms is governed by a multitude of traits (Bohan et al., 2019; Farrell et al., 2020). The use of selection index theory (Hazel, 1943) to generate a single value of the genetic merit of an animal, is commonly used across farmed species including sheep (Conington et al., 2004; Swan et al., 2007; Santos et al., 2015), goats (Ziadi et al., 2021), beef (Twomey et al., 2020), and dairy (Miglior et al., 2005). Rates of genetic gain or response to selection are commonly modeled for individual traits for sheep (Swan et al., 2007; Casellas et al., 2015; Santos et al., 2017), less commonly reported in the literature is the validation of breeding objectives (Connolly et al., 2016; Twomey et al., 2020), this is especially true for sheep, where validation studies have tended to focus on specific traits (Conington et al., 1998) or on controlled experiments (Márquez et al., 2012; Fetherstone et al., 2021a, 2021b). Access to a national database, such as was available in the present study allows, firstly large numbers of animals to be accessed per trait (range 9,197 to 47,169 in the present study) and secondly allows animals to be compared in a commercial environment. However, despite the differences in approach between the current and previous validation studies, the results are consistent in showing the use of breeding indexes resulted in an improvement in animal performance in some pertinent traits including lamb live weights (Márquez et al., 2013), and number of lambs born and weaned (Fetherstone et al., 2021b). The continuous validation of breeding objectives is not only of importance for geneticists, to ensure their products yield the anticipated results, but also for sheep producers, as without their continued buy-in breeding objectives become obsolete. Given the low number of progeny per sire, coupled with the low to moderate heritability associated with the traits investigated in the present study, moderate to low accuracy values were associated with both the maternal (average 52%) and terminal (average 69%) national breeding objectives. This can result in larger index or trait movement on individual animals and can therefore erode producer’s confidence in the breeding objectives. Results from this study, however, highlight that, on average, selecting animals based on either the maternal or terminal breeding index will result in superior performance across a plethora of economically important traits.

Within the Irish national sheep breeding program two breeding objectives, encompassing different suites of traits and relative weightings, have been developed. The terminal breeding objective focuses primarily on breeding animals that are destined for slaughter; in contrast, the maternal breeding objective focuses on breeding female replacements with favorable maternal attributes whilst also taking cognizance that a large proportion of her progeny may be destined for slaughter. Given the disparity between both breeding objectives, it is unsurprising that the magnitude of difference in phenotypic performance investigated in this study differed depending on the breeding objective under investigation. For example, a difference of 0.98% in perinatal lamb survival was observed between lambs born to ewes of high and low maternal index, however, perinatal lamb survival did not differ based on the terminal index of the sire. The difference in the association between perinatal lamb survival and both breeding objectives may be due to the inclusion of both a direct and maternal EBV for lamb survival in the maternal index, whereas only the direct EBV for perinatal lamb survival is included in the terminal index. However, results for the present study showed that selecting sires on the terminal index had no (negative) impact on lambing performance traits. This in itself is noteworthy given that the terminal index is selecting for heavier lambs (at least from preweaning onwards), coupled with the negative relationship reported between lamb (birth) weight and lambing difficulty and dystocia (McHugh et al., 2020) suggests that the terminal index is improving lamb live weight performance without dis-improving lambing performance. Greater differences in lamb live weight between animals of high and low genetic merit were observed from birth through to the postweaning period based on the maternal index in comparison to the terminal index. This may be partly explained by the maternal milk effect of the ewe, which corroborates results published by Massender et al. (2019) which showed a significant maternal heritability for lamb live weight from birth to postweaning. However, a greater difference in age at slaughter was observed between animals of high and low genetic merit based on the terminal index (8.34 d) compared to the maternal index (4.45 d), suggesting that the terminal index is selected strongly for lamb postweaning growth potential. The magnitude of the differences between animals of high and low index in the present study was greater than those reported by Fetherstone et al. (2021a) for traits including lamb survival and number of lambs born, but very similar in terms of lamb live weights pre and weaning to those reported previously by Márquez et al. (2012) at both 5 (+0.3 kg) and 10 (+0.5 kg) weeks of age.

The number of lambs born and reared has been highlighted as one of the key drivers in flock productivity and profitability both nationally (Bohan et al., 2019) and internationally (Farrell et al., 2020), however, despite this, the average weaning rate per ewe joined has remained relatively low or static in Ireland (Bohan et al., 2017). Results from the present study indicate that ewes ranked high on the maternal index, on average, produce an additional 0.17 lambs per lambing event in comparison to ewes ranked as low. This coupled with the greater rates of perinatal lamb survival associated with lambs born to ewes ranked high on the maternal index indicates that a clear strategy through the selection of animals based on their genetic merit is now available to the Irish sheep industry to increase the number of lambs weaned per ewe of the national population.

Differences were not detected or no clear trend was observed across the five strata investigated in the current study on either the maternal or terminal indexes in a number of different parameters including health traits (lameness and dag score), carcass characteristics (conformation and fat score) and ewe barren rate (maternal breeding objective only). These traits (i.e., health, carcass, and barren rate) have been introduced to the national breeding objectives relatively recently (O’Brien et al., 2017) and therefore tend to have a relatively low number of records recorded to date; this coupled with the low relative emphases placed on these traits (Bohan et al., 2019), may help to explain the lack of divergence observed in these traits. For these traits, secondary analyses were undertaken whereby the association between the individual trait EBV and the corresponding phenotypic performance were investigated, and in most incidences, differences (*P* < 0.01) were observed (results not presented). For example, a (favorable) 1.25% difference in perinatal lamb survival was observed between animals in the top and bottom 20% EBV for perinatal lamb survival; similarly, a 7.61% and 2.27% difference was observed in the proportion of lambs that required assistance at lambing for the EBV for single and multiple lambing ease, respectively. These results highlight that the genetic evaluations that underpin the breeding objectives are yielding favorable results but that the breeding objectives may require some additional research, including the potential revision of the economic weightings assigned to these traits. This also suggests if producers are selecting an animal based on a particular trait such as health or ewe barren rate, then they should focus on the animal’s individual EBV in combination with the overall breeding objective.

Results from this study highlight that selecting animals based on either the maternal or terminal breeding objective will simultaneously result in greater animal performance and productivity for a number of pertinent traits. Although the magnitude of differences on individual traits observed in the present study was generally small the accumulation of these differences to overall farm profitability warrants further investigation to assess the true potential of the national genetic evaluations to improve the national flock productivity and profitability.

## Supplementary Material

txac099_suppl_Supplementary_Appendix
