# Supplementary material for: Validation of maternal and terminal sheep breeding objectives using Irish field data
Source: Transl Anim Sci. 2022 Jul 23;6(3):txac099. doi: 10.1093/tas/txac099 (PMC9391924; doi:10.1093/tas/txac099)
Supplement: txac099_suppl_Supplementary_Appendix [file txac099_suppl_supplementary_appendix.docx]

**APPENDIX 1:** Formula used to calculate the maternal and terminal index of all animals.

Maternal Index **=** €1.00*EBV_NLB_ + €0.36*EBV_SURV_ + €0.002*EBV_LES_ + €0.002*EBV_LEM_ - €0.16*EBV_BAR_ + €1.11*EBV_MWT_ - €0.27*EBV_EWEWT_ + €0.49*EBV_MSURV_ + €0.003*EBV_MLES_ + €0.002*EBV_MLEM_ -€0.13*EBV_DTS_ + €1.89*EBV_CONF_ - €0.43*EBV_FAT_ - €0.20*EBV_DAG_ - €0.05*EBV_LAML -_€0.11*EBV_LAME_

Terminal Index = €0.29*EBV_SURV_ + €0.001*EBV_LES_ + €0.001*EBV_LEM_ -€0.10*EBV_DTS_ + €1.55*EBV_CONF -_ €0.35*EBV_FAT_ - €0.15*EBV_DAG_

- €0.04*EBV_LAML_

where EBV_NLB_ is the estimated breeding values (EBV) for number of lambs born, EBV_SURV_ is the EBV for lamb survival, EBV_LES_ is the EBV for lambing ease for singleton lambs, EBV_LEM_ is EBV for lambing ease for multiple lambs, EBV_BAR_ is the EBV for ewe barren rate, EBV_MWT_ is the EBV for maternal pre weaning weight, EBV_EWEWT_ is the EBV for ewe weight, EBV_MSURV_ is the EBV for maternal lamb survival, EBV_MLES_ is the EBV for maternal lambing ease for singleton lambs, EBV_MLEM_ is the EBV for maternal lambing ease for multiple lambs, EBV_DTS_ is the EBV for ages to slaughter, EBV_CONF_ is the EBV for carcass conformation, EBV_FAT_ is the EBV for carcass fat, EBV_DAG_ is the EBV for dag score, EBV_LAML_ is the EBV for lameness in lambs and EBV_LAME_ is the EBV for lameness in ewes.
